# How effective are CBT and CBT‐based interventions in Type 1 and Type 2 diabetes? An umbrella review

**DOI:** 10.1111/dme.70271

**Published:** 2026-02-20

**Authors:** Hanna S. Press, Leeanne Nicklas

**Affiliations:** ^1^ School of Medicine University of Dundee Dundee UK

**Keywords:** anxiety, cognitive behavioural therapy, depression, diabetes mellitus, psychological distress, review

## Abstract

**Aims:**

Significant evidence base supports cognitive behavioural therapy (CBT) for diabetes. Large variations in practice make it difficult to assess the impact of different types of delivery. This umbrella review aimed to synthesise evidence on the effectiveness of CBT and CBT‐based interventions for diabetes‐related distress, comorbid depressive symptoms, anxiety symptoms and HbA1c levels. A secondary aim investigated whether effectiveness differs in CBT delivered by a CBT therapist compared to CBT‐based interventions that are delivered by multidisciplinary staff.

**Methods:**

Databases like CINAHL, MEDLINE, PsychINFO, PsychArticles and Cochrane Library were searched for systematic reviews and meta‐analyses between 2014 and 2023. Review quality was assessed using the Scottish Intercollegiate Guidelines Network checklist. For the secondary aim, findings of randomised controlled trials were distinguished within reviews.

**Results:**

Eleven systematic reviews and meta‐analyses were included. Four of the reviews were rated as ‘high’‐quality reviews, five as ‘acceptable’ and two as ‘low’‐quality reviews. CBT‐based interventions delivered by multidisciplinary staff significantly reduced depressive symptoms, diabetes‐related distress and HbA1c levels. CBT therapy delivered by CBT therapists was associated with significant reductions in diabetes‐related distress, anxiety symptoms, depressive symptoms and HbA1c levels.

**Conclusions:**

CBT and CBT‐based interventions were similarly effective for depressive symptoms and HbA1c levels. Anxiety symptoms only improved following CBT delivered by CBT therapists, while diabetes‐related distress reduced more than for CBT‐based interventions. Significant heterogeneity and variation in quality in reviews mean that further research is required.

**What's new?**

**What is already known?**

Cognitive behavioural therapy (CBT) is broadly effective in diabetes, but variation in the definition of CBT and CBT‐based interventions means it is difficult to translate effectiveness findings into practice guidance.

**What this study has found?**

- CBT‐based interventions provided by multidisciplinary staff significantly reduced depressive symptoms, diabetes‐related distress and HbA1c levels.
- CBT interventions delivered by a CBT therapist were more effective at reducing diabetes‐related distress and anxiety symptoms.
- Depressive symptoms and HbA1c improvements were similar across delivery types.

**What are the implications of the study?**

- This review compared physical and mental health outcomes associated with CBT and CBT‐based interventions, providing clarification on the outcomes associated with these types of delivery to meet the needs of different presentations in practice.

## 1 INTRODUCTION

ICD‐11 defines diabetes mellitus as a chronic metabolic disease in which deficiencies in insulin action and/or insulin secretion result in elevated blood glucose levels and disturbances in the metabolism of carbohydrates, fats and protein.^1^ Prevalence is rising, with over 537 million adults recorded with diabetes in 2021, with an expected increase to 783 million adults by 2045.^2^

Diabetes is associated with physical and psychological challenges. Self‐management of blood glucose levels is important in reducing long‐term health consequences in Type 1 and Type 2 diabetes.^3^ Measured as glycosylated haemoglobin A1c levels (HbA1c), this indicates average blood glucose levels of the previous 3 months, although around 70% of individuals with Type 1 diabetes and one third of individuals with Type 2 diabetes do not reach their target HbA1c levels.^3^ Difficulties managing diabetes are common, as re‐establishing glycaemic control requires complex and constant self‐management behaviours across diet, medication requirements and more, while benefits can only be seen months later.^4^ Psychological interventions have been offered to improve self‐management behaviours that influence daily glucose regulation, with sustained behavioural changes reflected in HbA1c levels commonly reported in the literature.^4^, ^5^ However, given the multiple factors affecting glycaemic control, psychological therapies are more likely to aim to improve psychological factors such as mental health difficulties in individuals with diabetes including depressive symptoms, anxiety symptoms and diabetes‐related distress, and this has increasingly been evaluated in trials.

Diabetes‐related distress (DRD) refers to distress individuals may experience following their diagnosis and worries about diabetes management, health complications and access to care and support.^6^ It is associated with reduced psychological well‐being, medication adherence and self‐management behaviours,^7^ as well as poor glycaemic control and reduced quality of life.^8^ DRD evidences a 40% prevalence^9^ and is typically measured using the Problem Areas in Diabetes (PAID)^10^ and Diabetes Distress Scale (DDS)^6^ to distinguish from general stress and comorbid depressive symptoms.

Mental health disorders are common comorbidities in diabetes, with depressive symptoms occurring twice as often as for people without diabetes^5^ and a 14% prevalence of anxiety symptoms disorders.^11^ Comorbid depressive symptoms have been suggested to impair diabetes management and self‐care, evidencing poorer glycaemic control, increased risk of health‐related complications and reduced life expectancy.^12^ Similarly, anxiety symptoms have been associated with increased risk of diabetes complications, depressive symptoms and reduced self‐care.^13^ People with diabetes and comorbid mental health issues have evidenced lower quality of life than individuals without diabetes,^14^ leading to reduced self‐management and functional impairment.

The impact of diabetes on well‐being, mental health difficulties, reduced glycaemic control and quality of life are associated with an increase in healthcare use and healthcare costs.^15^ The high prevalence of diabetes in addition to the personal and economic costs of these outcomes highlights a need for effective interventions.

### 1.1 Psychological interventions

Psychological therapies, specifically cognitive behavioural therapy (CBT), are recommended by NICE^16^ as a first‐line treatment for comorbid depressive symptoms and anxiety symptoms across long‐term physical health conditions such as diabetes. A stepped care model recommends low‐intensity self‐help programmes for mild to moderate depressive symptoms and high‐intensity CBT (individual or group‐based) for moderate‐to‐severe depressive symptoms.^16^ Within high‐intensity CBT for depressive symptoms, outcomes are equivalent between individual and group delivery.^17^ The Matrix^18^ guides the delivery of psychological therapies in Scotland and summarises efficacy evidence for Type 1 and Type 2 diabetes across various outcomes. The Matrix recommends low‐intensity interventions, such as guided CBT‐based self‐help (often delivered by multidisciplinary health professionals who have additional training in the intervention) and computerised CBT for people with mild‐to‐moderate depressive symptoms and DRD. However, this recommendation is based on a weak evidence base, suggesting that further investigations into low‐intensity interventions for diabetes are necessary. Individual CBT is recommended as a high‐intensity intervention for moderate‐to‐severe mental health issues to improve glycaemic control, depressive symptoms and DRD. These recommendations are based on high‐quality systematic reviews or randomised controlled trials (RCTs); however, both NICE^16^ and Matrix^18^ guidelines are based on evidence prior to 2014. Considering the growing evidence base for CBT in diabetes,^19^, ^20^ a review reflecting more recent published literature is required.

There are several existing systematic reviews and meta‐analyses covering and supporting the general effectiveness of CBT for depressive symptoms, DRD and glycaemic control in individuals with Type 1 or Type 2 diabetes since 2014.^4^, ^19^, ^20^, ^21^ Interestingly, most reviews do not differentiate Type 1 and Type 2 diabetes within their analyses.^19^ The rationale for this is the proposed similarity across physical and psychological symptoms for both diabetes types^21^ despite differing aetiology and prevalence. As typical in the efficacy literature for psychological therapies, psychometric questionnaires are used to assess changes in depressive symptoms and anxiety symptoms. While there are a range of different measures in studies, most use validated measures that have standardised estimates of what constitutes reliable improvement. Many reviews focus on depressive symptoms as a primary outcome,^22^ reviews also examine DRD^20^ and HbA1c levels.^4^, ^21^ Although anxiety symptoms are also prevalent in diabetes,^11^ it is mostly considered a secondary outcome within the CBT‐efficacy literature.^23^, ^24^

### 1.2 Heterogeneity in definitions of CBT

While there is a general sense of CBT as an established evidence‐based approach, there is unhelpful variation in the definition of ‘cognitive behavioural therapy’ within diabetes reviews. As an example, Yang et al.^25^ suggested CBT‐based interventions are effective in improving glycaemic control and depressive symptoms but included a wide range of therapies in their definition, including therapies that have their own protocols such as mindfulness‐based CBT or Acceptance and Commitment Therapy. The term CBT in the published literature in diabetes varies in terms of healthcare provider, content, duration and format; however, this is often not distinguished within reviews.^19^, ^26^, ^27^, ^28^ This can make it difficult to draw conclusions for the effectiveness of CBT in diabetes.

CBT is traditionally delivered by trained mental health professionals in individual or group format of between 8 and 16 sessions. Essential components of CBT include techniques targeting unhelpful beliefs (cognitions) and behaviours. In specialist CBT, the intervention is guided by a psychological formulation of the person's difficulties, highlighting which unhelpful beliefs and behaviours are addressed within therapy and through activities between sessions. As mental health difficulties in long‐term conditions are common but often unrecognised, multidisciplinary healthcare providers in direct contact with people with diabetes have been supported to provide psychological or CBT‐based techniques with some additional training.^29^ CBT can then be provided by psychologically trained mental health specialists if required.

The involvement of multidisciplinary staff^29^ expanded alongside developments of the ‘Improving Access to Psychological Therapies programme’ (IAPT)^30^ and differentiated using the terms low‐ and high‐intensity delivery of CBT. As outlined in the NICE^16^ and Matrix^18^ guidance, low‐intensity CBT refers to self‐help or psychoeducation groups delivered by multidisciplinary staff, while high‐intensity CBT refers to CBT delivered by trained professionals.^31^ More recently, NHS Scotland^32^ proposed a competency‐based framework, distinguishing specialist, enhanced, skilled and psychologically informed psychological practice. Originally developed for trauma work, this model has been expanded to a range of professional practice, including those providing CBT and CBT‐based interventions for long‐term health conditions such as diabetes. With specialist and enhanced practice being akin to low‐ and high‐intensity terminology, specialist practice refers to formulation driven CBT therapy often conducted individually by a psychologically trained CBT therapist. Enhanced practice refers to low‐intensity (protocol‐driven or manualised) psychological interventions. These are delivered by professionals with additional training and involve quality assurance mechanisms, such as clinical supervision, along with mechanisms for checking fidelity. Skilled practice refers to multidisciplinary staff providing CBT‐based skills within their routine care (e.g., psychoeducational groups, behavioural change techniques). Both low intensity and skill‐based interventions are often group‐based. Psychologically informed care refers to baseline psychological knowledge in all healthcare workers.

Considering these significant differences between practice types in terms of content and delivery, it is likely CBT and CBT‐based interventions may differ in their effectiveness across outcomes in diabetes. For instance, CBT delivered in line with specialist practice^27^ showed significant changes in depressive symptom scores in people with diabetes, whereas a CBT‐based intervention delivered by nurses trained in CBT skills (the Diabetes‐6 trial) demonstrated no significant improvements in depressive symptoms.^28^ However, this paper raised another important aspect that should be considered in the delivery of psychological interventions, namely whether the intervention is being delivered as intended. In the Diabetes‐6 trial, later fidelity checks identified no differences between the active and control interventions as nurses were also implementing CBT skills into usual care.^33^ This highlights the importance of distinguishing psychological practice when assessing CBT effectiveness.

While there is considerable evidence on the use of CBT and CBT‐based interventions in diabetes, this has not been synthesised since the NICE^16^ and Matrix^18^ guidelines, suggesting the need for a review to reflect the literature published since 2014. Moreover, large variations in psychological practice (CBT vs. CBT‐based and provider) make it difficult to assess the impact of different types of delivery for the effectiveness of CBT in diabetes and the recommendations made for clinical practice. Therefore, the current umbrella review aims to answer the following questions:

1. Does the literature since 2014 confirm or change our understanding of the effectiveness of CBT and CBT‐based interventions for diabetes‐related distress, depressive symptoms, anxiety symptoms and HbA1c levels in Type 1 and Type 2 diabetes outlined in clinical guidance?
2. Does CBT conducted through specialist practice differ from CBT‐based interventions conducted through enhanced or skilled practice in their effectiveness for diabetes‐related distress, depressive symptoms, anxiety symptoms and HbA1c levels?

## 2 METHODS

### 2.1 Search strategy

A multi‐phase search was adopted to capture most of the relevant literature.^34^ An initial key word search examined the use of CBT for well‐being outcomes in individuals with diabetes mellitus, then, a population, intervention, comparison and outcome (PICO)^35^ framework was established to guide finalised research questions and search strategy (Table 1). As the preliminary search revealed a well‐established evidence base, the search strategy focused on the highest level of evidence i.e., systematic reviews and meta‐analyses of RCTs. Initial searches focused on Type 2 diabetes to reduce variation, however this was extended to include Type 1 diabetes, as most reviews combined Type 1 and Type 2 diabetes in their analyses.

**TABLE 1:** PICO framework.

P—Patient or population	Adults (18 years or above) with a diagnosis of type 1 or 2 diabetes mellitus
I—Intervention	CBT or CBT‐based interventions (individual or group format)
C—Comparison	Treatment as usual, waitlist controls, diabetes education, active controls (other non‐CBT‐based psychological interventions or pharmacological interventions)
O—Outcome	Psychological—diabetes‐related distress, depressive symptoms, anxiety symptoms Physiological—glycaemic control (HbA1c level)

#### 2.1.1 Literature search

**FIGURE 1:**
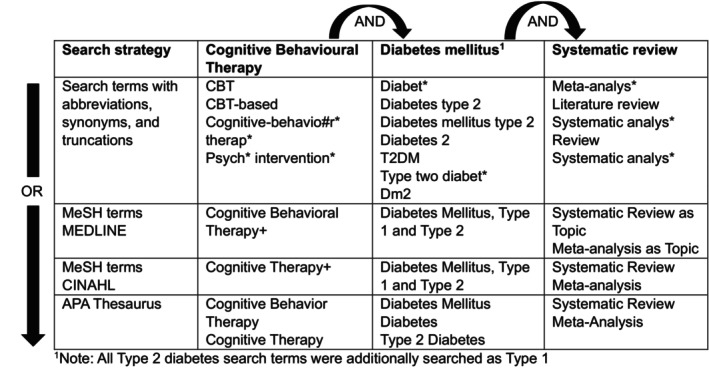
Search terms.

To identify relevant systematic reviews and meta‐analyses, five databases were searched between June and July 2023: CINAHL, Cochrane Library, MEDLINE, APA PsychINFO and APA PsychArticles. Three underlying concepts were identified as the most relevant terms in each database: cognitive behavioural therapy, diabetes mellitus types 1 and 2, and systematic reviews. Research design was included as a search term as most reviews were not registered in methodology database limiters. Searching title, abstract and keywords, search terms (Figure 1) with abbreviations, synonyms and truncations to consider spelling differences were combined using Boolean operators (AND/OR). Medical Subject Headings terms (MeSH) and APA Thesaurus of psychological index terms were used. Database limiters were set for English article language and publication year, only including reviews published after the last Matrix update in 2014.^18^ Supplementary searches included manually searching reference lists to identify any missed reviews.

**FIGURE 2:**
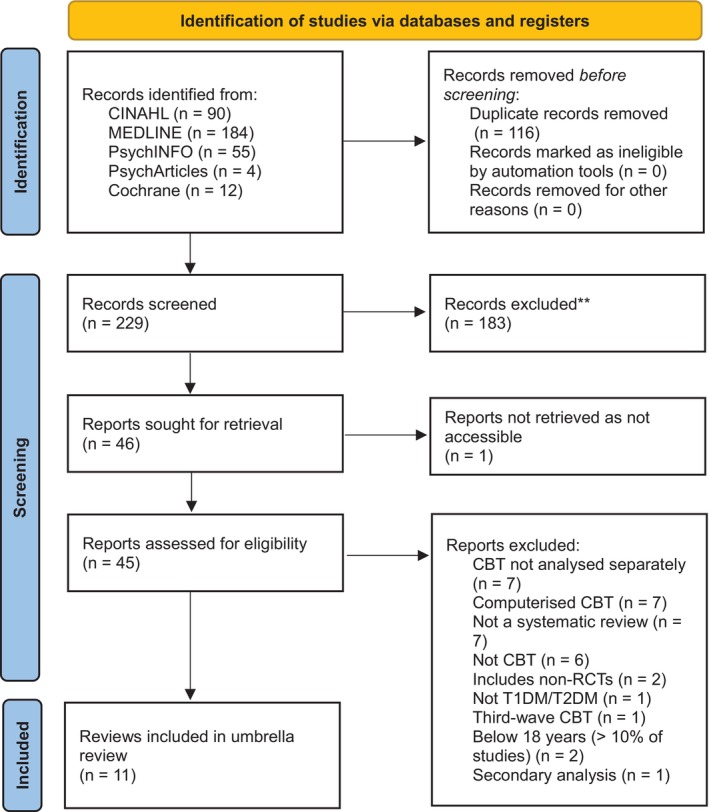
PRISMA flowchart.

The initial literature search resulted in a total of 345 articles, which were downloaded to a reference management software (RefWorks), where duplicates (*n* = 116) were removed. Supplementary searches identified no further articles. Screening of titles and abstracts, as well as the lack of accessibility to one review, led to the retrieval and full‐text evaluation of 45 citations. A total of 11 citations met the inclusion criteria and were selected for this umbrella review. The reasons for exclusion were most commonly outcomes for CBT not being analysed separately, not systematic review, not CBT and computerised CBT. Figure 2 depicts the PRISMA flowchart of the selection process.

### 2.2 Inclusion criteria

For inclusion in this umbrella review, systematic reviews and meta‐analyses were required to:

1. Focus solely on RCTs.
2. Include women or men participants with a diagnosis of Type 1 or Type 2 diabetes.
3. Have at least 90% of participants with a minimum age of 18 years.
4. Focus on CBT or CBT‐based interventions or clearly distinguish these from other psychological interventions. This is defined as interventions with a cognitive and behavioural element delivered by a mental health or health care professional and with no limitations on delivery, format or duration.
5. Include comparators such as treatment as usual, waitlist controls, diabetes education or other active non‐CBT controls.
6. Examine at least one of the pre‐specified outcomes as a primary or secondary outcome: diabetes‐related distress, depressive symptoms, anxiety symptoms and HbA1c level.
7. Include outcomes assessed using validated and reliable psychometric measures, such as the DDS^6^ or PAID^10^ for DRD. Further examples include the Beck Depression Inventory II (BDI‐II)^36^ and the Generalised Anxiety Disorder Scale (GAD‐7).^37^ A change in glycaemic control was measured by HbA1c levels.

### 2.3 Exclusion criteria

Systematic reviews and meta‐analyses were excluded if they:

1. Focused on participants with gestational diabetes or pre‐diabetes.
2. Included participants' complex comorbidities, e.g., Axis II disorders.
3. Focused solely on computerised CBT (no health care professional involvement) or third‐wave therapy (ACT/mindfulness‐based CBT).
4. Were published in a language other than English or prior to 2014.

As a secondary objective, the current umbrella review aimed to distinguish interventions delivered in line with specialist practice from interventions delivered through enhanced or skilled psychological practice. This reduces variation, increasing confidence and transferability of current findings to clinical practice. As reviews typically did not make a distinction, this information was either drawn from descriptions of the intervention or, if insufficient, the original RCTs and findings related to specialist practice (CBT delivered by a therapist) were extracted from the reviews and examined separately. Group CBT‐based interventions delivered by multidisciplinary staff (enhanced and skilled practice) were not further differentiated. Pooled effect sizes were narratively synthesised from the findings of included reviews where available.

### 2.4 Quality appraisal

The quality of each included review was appraised by the Scottish Intercollegiate Guidelines Network methodology checklist for systematic reviews and meta‐analyses.^38^ The checklist comprises 12 areas of internal validity before the overall assessment as high, acceptable or low quality: a clear PICO framework, exhaustive literature search, data selection and extraction by two researchers, publication status, excluded studies, detailed study characteristics, quality appraisal and appropriate consideration of this. Appropriate methodology to combine findings, publication bias and conflicts of interest are also assessed within the checklist.

Methodological quality was primarily assessed by one reviewer; this limitation was partially addressed by involvement from a second reviewer who independently rated a random six of 11 included reviews. Cohen's Kappa was calculated to identify inter‐rater agreement,^39^ finding substantial inter‐rater agreement of 0.67. Areas of disagreement were identified as whether appropriate methods were used to combine individual study findings, appropriate assessment of publication bias, and whether conflicts of interest were declared. Disagreements were resolved through discussion and re‐examining the reviews.

## 3 RESULTS

### 3.1 Review characteristics

Eleven reviews which met the inclusion criteria for this umbrella review were identified (Figure 2). Five of these were conducted in the UK, four in China, one in Indonesia and one in Australia (Table S1 in Appendix S1). Most reviews combined studies investigating Type 1 and Type 2 diabetes (*n* = 8), while three investigated only Type 2 diabetes. Three reviews included studies with a specific focus on diabetes and comorbid depressive symptoms.^19^, ^21^, ^22^ Four reviews included studies that examined depressive symptoms as the primary outcome,^19^, ^22^, ^40^, ^41^ five reviews focused on HbA1c as the primary outcome^4^, ^21^, ^23^, ^26^, ^42^ and one review examined DRD as a primary or secondary outcome within included studies.^20^ One additional review prioritised self‐care behaviour as the primary outcome, with depressive symptoms, anxiety symptoms and HbA1c as secondary outcomes.^24^ The total number of participants within reviews varied between 834^40^ and 18,659^4^ and mean ages ranged from 34.2 years^4^ to 70.7 years.^20^

Six reviews exclusively included studies utilising CBT. One review conducted separate analyses for traditional and third‐wave CBT,^20^ while four reviews included various psychological interventions, differentiating CBT in sub‐analyses.^4^, ^22^, ^41^, ^42^ The type of CBT intervention varied widely. While sessions were mostly conducted in a face‐to‐face format (individual, group), session length (15–120 min) and treatment duration (5 days–2 years) differed considerably. Reviews did not differentiate between types of psychological practice and interventions were conducted by CBT therapists (psychologists and other professionals with formal CBT training) or multidisciplinary staff with some training in CBT skills (diabetes nurses, dieticians, etc.). Only one review specified that the CBT interventions were non‐specialist delivered,^42^ while for other reviews this information was drawn from intervention descriptions outlined in their findings. An overview of review findings is presented in Table S1, and an in‐depth analysis of the outcomes for CBT delivered in various formats in Table 2.

**TABLE 2:** Summary of findings for different types of CBT practice.

Type of psychological practice	Diabetes‐related distress	Depressive symptoms	Anxiety symptoms	HbA1c
Specialist practice (e.g., individual CBT delivered by a CBT therapist)	*Post‐treatment*: Moderate effect (*d* = 0.57, *p* = 0.04).^43^ Effect size not calculated, significant reduction.^27^, ^44^ *Long‐term*: 9‐month follow‐up moderate effect (*d* = 0.35, *p* = 0.002).^45^	*Post‐treatment*: Large effect (*d* = 1.00–1.49, *p* < 0.001–0.02).^43^, ^46^ Effect sizes not calculated, significant reduction in mean scores.^27^, ^47^ 85% remission rate (*p* < 0.0001).^49^ No significant reduction (*p* = 0.12–0.26).^44^, ^48^ *Long‐term*: Moderate effect at 9 months (*d* = 0.62, *p* < 0.001).^45^ 70% remission rate after 6 months.^49^ Non‐significant effects at follow‐ups 4–12 months post‐treatment (*p* = 0.10).^47^, ^50^	*Post‐treatment*: Large effect (*d* = 0.82–0.88, *p* = 0.01–0.04).^43^, ^46^ *Long‐term*: 9‐month follow‐up moderate‐to‐large effect (*d* = 0.78; *p* < 0.001).^45^	*Post‐treatment*: Effect sizes not calculated, significant reduction in mean scores.^44^, ^47^ Non‐significant findings (*p* = 0.38–0.89),^27^, ^43^, ^49^, ^S51^ *Long‐term*: Maintenance of gains at 8‐month follow‐up.^47^ Significant improvement at 6 months.^49^ Non‐significant small effect (*d* = 0.15, *p* = 0.38).^45^
Enhanced/skilled practice (e.g., group approaches delivered by multidisciplinary professionals with or w/o psychological therapist)	*Post‐treatment*: Small effect (SMD = −0.149, 95% CI −0.276 to −0.023, and *d* = 0.275, *p* = 0.021–0.014), (*I* ^2^ = 54.8%).^19^, ^20^ ^respectively^ DRD as primary outcome (SMD = −0.278, 95% CI −0.488 to −0.068, *p* = 0.010) (*I* ^2^ = 62.8%).^20^ Pooled effect estimates not calculated, significant improvement,^21^ mixed findings.^24^ No improvements (*p* = 0.05),^22^ non‐significant findings (*p* > 0.05).^42^ *Long‐term*: Pooled effect estimates not calculated, mixed findings 4–12 months post‐intervention.^21^	*Post‐treatment*: Moderate‐to‐large effect (*d* = 0.301, SMD = −0.43 to −1.22, *p* < 0.00001–0.01), (*I* ^2^ = 23–92%).^19^, ^20^, ^21^, ^22^, ^23^, ^40^ Pooled effect estimates not calculated, mixed findings.^24^, ^42^ 17% recovery rate.^41^ *Long‐term*: Moderate effect at 6 months (SMD = −0.43, *p* = 0.02) and at 12 months small to moderate effect (SMD = −0.26 to −0.38, *p* = <0.0001–0.001), (*I* ^2^ = 25–73%).^21^, ^40^ Pooled effect estimates not calculated, non‐significance at 12 months (*p* = 0.09–0.70).^42^	*Post‐treatment*: Moderate‐to‐large effect (SMD = −0.41 to −1.03, *g* = 0.72, *p* < 0.0001–0.02), (*I* ^2^ = 0–85%).^21^, ^23^ Moderate effect, 1 study (*g* = 0.72, *p* = 0.002).^20^ Non‐significant findings (*p* = 0.275–0.46).^19^, ^24^ Overall non‐significant findings (*p* = 0.47), moderate effect only when CBT duration >6 months (SMD = −0.49, *p* = 0.01).^22^ *Long‐term*: Moderate effect at 6 months (SMD = −0.56, *p* = 0.0002) (*I* ^2^ not provided), but non‐significance at 12 months based on 1 study (*p* = 0.16).^21^	*Post‐treatment*: Small effect (SMD = −0.25, 95% CI −0.42 to −0.09; *I* ^2^ = 54%; ≈4 mmol/mol reduction (−0.44% change))^4^; Large effect (SMD = −0.97, 95% CI −1.37 to −0.57; *I* ^2^ = 96%)^23^; Significant reduction (SMD = −26 mmol/mol (−0.2%), *p* = 0.07), (*I* ^2^ = 0%).^21^ Pooled effect estimates not calculated, mixed findings (*n* = 4).^24^ Non‐significant findings (*p* = 0.15–0.812), (*I* ^2^ = 0–74%).^19^, ^20^, ^22^, ^26^, ^42^ *Long‐term*: Increased effect up to 8 months (SMD = −28 mmol/mol (−0.4%), *p* = 0.0001), (*I* ^2^ = 44%), non‐significant 12 months, 1 study (*p* = 0.18).^21^ Moderate effect at follow‐up >6 months (*MD* = −0.43, *p* < 0.00001), (*I* ^2^ = 70%).^26^

### 3.2 Quality appraisal

Using the SIGN checklist for systematic reviews and meta‐analyses,^38^ four of the reviews were rated as ‘high quality’ with little or no risk of bias, five as ‘acceptable’ with most criteria fulfilled and two as ‘low quality’ with significant flaws in study design. These are summarised in Table 3 with key limitations highlighted. While it is recommended to limit analyses to moderate and low risk of bias studies, the small number of studies within this review meant no examined reviews were excluded. Possible differences in results or effect sizes between high and low risk of bias were evaluated (Appendix S2 for detailed quality appraisal).

**TABLE 3:** Quality of included reviews using SIGN checklist.

Review	Overall quality rating score*	Notes/issues
An et al. (2023)^19^	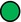	
Dong et al. (2023)^26^	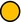	Publication bias and high risk of bias in 2 studies not considered when discussing results
Fiqri et al. (2022)^24^	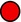	No supplementary searches, no sample characteristics provided. Reporting and discussion of results does not coincide with study statistics in tables
Jenkinson et al. (2022)^20^	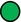	
Mather et al. (2022)^41^	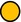	No consideration of high risk of bias studies when discussing results
Oyedeji et al. (2022)^42^	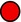	No use of a standardised quality appraisal tool. Summary range for quality scoring across all studies is not acceptable
Winkley et al. (2020)^4^	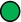	
Li et al. (2017)^22^	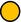	No supplementary searches
Uchendu and Blake (2017)^21^	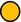	Characteristics summarised across studies
Wang et al. (2017)^40^	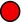	Only searched 2 databases, no details on data extraction methods and limited description of the interventions included
Chapman et al. (2015)^23^	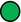	

* Quality rating score: 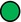
—High, 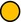 **—**Acceptable, 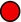
—Low.

### 3.3 Clinical outcomes

Table 2 details a summary of findings for all different types of CBT practice across DRD, depressive symptoms, anxiety symptoms and HbA1c levels in individuals with Type 1 or Type 2 diabetes. Outcomes are presented from findings of reviews that grouped different types of CBT delivery together. This review then extracted information from 10 RCTs within the examined reviews that employed CBT delivered by specialist practice. See Table S2 in Appendix S1 for study characteristics and the focus of the CBT intervention.

#### 3.3.1 Diabetes‐related distress

##### Results for all CBT‐based interventions in reviews

Six reviews considered the effects of CBT‐based interventions delivered by a range of professionals (enhanced or skilled practice) on DRD. Results were variable. A small effect was described in two high‐quality reviews,^19^, ^20^ one of which had moderate statistical heterogeneity.^20^ Two further reviews with low^24^ and acceptable^21^ quality ratings were unable to pool findings due to the variety in outcome measures due to the reporting of mixed findings for DRD^24^ or due to showing significant improvement post‐treatment but mixed findings 4–12 months post‐intervention.^21^ An acceptable quality review, Li et al.,^22^ found a significant lack of improvement post‐treatment, while reductions in DRD for non‐specialist delivered CBT‐based interventions did not reach significance.^42^

##### Cognitive behavioural therapy RCTs

CBT delivered through specialist practice was evaluated in four RCTs. Individual CBT delivered by a CBT therapist was shown to effectively reduce DRD in the RCT studies. A moderate effect was detected in one study using the PAID scale,^43^ while two studies utilising the DDS‐17 found significant improvements in mean scores.^27^, ^44^ Regimen‐related distress was also found to positively correlate with HbA1c levels.^44^ Only one study, a secondary analysis of Tovote et al.,^43^ examined long‐term impact on DRD, finding a small effect at 9 months follow‐up.^45^

#### 3.3.2 Depressive symptoms

##### Results for all CBT‐based interventions in reviews

Depressive outcomes were identified by nine reviews with most reviews (*n* = 6) demonstrating the effectiveness of CBT interventions delivered by multidisciplinary staff for depressive symptoms. A significant moderate‐to‐large effect across three high‐quality,^19^, ^20^, ^23^ two acceptable^21^, ^22^ and one low‐quality^30^ meta‐analyses can be synthesised. However, high statistical heterogeneity should be noted, impacting confidence in the findings. Treatment duration of 2–6 months was suggested to be more effective for comorbid depressive symptoms than longer CBT.^22^ Two low quality reviews, Fiqri et al.^24^ and Oyedeji et al.,^42^ described mixed results and were unable to pool findings, while an acceptable quality review Mather et al.^41^ suggested only a 17% recovery rate for people with diabetes. Furthermore, at 6 and 12 months post‐intervention, two reviews with acceptable^21^ and low‐quality^40^ ratings suggested a reduction in effect size, while the low‐quality review Oyedeji et al.^42^ suggested no maintenance of gains.

##### Cognitive behavioural therapy RCTs

Nine RCTs investigated CBT delivered through specialist psychological practice. Five of the nine RCTs evidenced significant improvements in depressive symptoms immediately post‐treatment. Two studies reported large effect sizes,^43^, ^46^ while two further studies showed significant reductions in mean depressive symptoms scores across various validated scales.^27^, ^47^ Lutes et al.^44^ and Higgins et al.^48^ found no significant improvements, possibly due to low baseline depressive symptoms scores in their sample. Moreover, Lustman et al.^49^ reported an 85% remission rate, which remained at 70% after 6 months. Other studies demonstrated a moderate effect at 6 and 9 months,^45^ or no significant improvements up to 12 months post‐treatment.^47^, ^50^

#### 3.3.3 Anxiety symptoms

##### Results for all CBT‐based interventions in reviews

Six reviews considered the effectiveness of CBT‐based interventions for anxiety symptoms with mixed findings. While two high‐quality reviews reported large effect sizes, they either evidenced high heterogeneity^23^ or based their results on one study.^20^ Uchendu and Blake,^21^ with an acceptable quality rating, found a moderate effect of CBT‐based interventions on anxiety symptoms, which was maintained at six but not 12 months. Lastly, three high,^19^ acceptable^22^ and low^24^ quality reviews suggested no overall improvements in anxiety symptoms following CBT‐based interventions, although Li et al.^22^ discovered a moderate effect when CBT was conducted for 6–12 months.

##### Cognitive behavioural therapy RCTs

Only three RCTs investigated the effectiveness of CBT delivered through specialist practice on anxiety symptoms. These did not specifically target anxiety symptoms or consider anxiety symptoms a primary outcome. The CBT approaches used were instead aimed at reducing depressive symptoms^43^, ^45^ or insomnia^46^ (Table S2 in Appendix S1 for further details). Two RCTs evidenced large effects for anxiety symptoms at the end of treatment,^43^, ^46^ while Tovote et al.^45^ found a moderate‐to‐large effect 9 months post‐intervention.

#### 3.3.4 HbA1c

##### Results for all CBT‐based interventions in reviews

Nine reviews considered the impact of CBT‐based interventions on HbA1c levels. These reviews included studies in which HbA1c was targeted as a primary or secondary outcome. Two high‐quality^4^, ^23^ and one acceptable quality^21^ reviews suggested improvements in HbA1c levels immediately post‐intervention. While Chapman et al.^23^ demonstrated a high effect size with high statistical heterogeneity, Uchendu and Blake^21^ found a smaller effect without statistical heterogeneity. This improvement was maintained after 6 but not 12 months. Five further reviews, including high,^19^, ^20^ acceptable^22^, ^26^ and low^42^ quality ratings, found no significant effect of CBT‐based interventions on HbA1c levels and one low quality review did not calculate pooled effects estimates, although one review^26^ evidenced a moderate effect long‐term i.e., more than 6 months after treatment.

##### Cognitive behavioural therapy RCTs

Seven RCTs included CBT delivered through specialist practice. In these studies, HbA1c was looked at as a secondary outcome. Two studies reported significantly improved HbA1c levels following CBT therapy.^44^, ^47^ Four further RCTs suggested no improvements in glycaemic control.,^27^, ^43^, ^49^, ^S51^ Long‐term findings are mixed, with maintenance of gains^47^ as well as non‐significance.^45^ Lustman et al.^49^ showed improved glycaemic control at 6 months post‐intervention, suggesting delayed effectiveness of CBT.

## 4 DISCUSSION

This umbrella review aimed to synthesise findings on the effectiveness of CBT and CBT‐based interventions for DRD, depressive symptoms, anxiety symptoms and glycaemic control, in individuals with Type 1 or Type 2 diabetes.

CBT‐based interventions significantly reduced diabetes‐related distress in three out of the six included reviews and effects were larger when treatment primarily targeted DRD.^20^ The only review investigating long‐term DRD was unable to pool results.^21^ The findings suggest a small improvement in DRD following CBT‐based interventions, however methodological issues with the included studies and the review methodology mean that this finding should be interpreted cautiously and further research investigating long‐term outcomes and including a larger number of studies is desirable.

For comorbid depressive symptoms, there appears to be a consensus across most meta‐analyses of a moderate‐to‐large symptom reduction following CBT‐based interventions (Table 2). The reviews that reported mixed effectiveness^24^, ^42^ are limited by low quality^42^ and having a small number of included studies.^24^ Effectiveness was larger in depression‐focused CBT, although smaller benefits were also found when targeting DRD,^20^ adding to previous literature on CBT effectiveness for chronic physical health conditions.^S52^ Despite this, statistical heterogeneity indicates significant clinical or methodological variation between studies,^S53^ warranting caution for implementation into clinical practice. With regards to the effectiveness of CBT‐based interventions for anxiety symptoms, methodological limitations provide a cautious conclusion of no overall improvement for anxiety symptoms. Large improvements in anxiety symptoms are limited by high heterogeneity,^23^ while other reviews base their conclusions on only one or two studies, preventing generalisations.^20^, ^21^, ^24^ A recent methodologically sound meta‐analysis suggested no symptom reduction for anxiety symptoms.^19^

Providing one possible reason for variation in depressive symptoms findings, Li et al.^22^ suggested shorter intervention duration (2–6 months) as more effective than longer duration. This may be due to an interaction effect between symptom severity and treatment duration.^S54^ High relapse rates of depression in diabetes^S55^ may also explain the reduced benefit of long treatment duration and the reduction in symptom improvement across time.^21^, ^40^ Interestingly, for anxiety symptoms, Li et al.^22^ suggested the overall non‐significant effect may become significant if treatment duration exceeded 6 months. Anxiety symptoms are often investigated as a secondary outcome in depressive symptoms‐focused CBT‐based interventions and may only reduce following the initial improvement in depressive symptoms. This is supported by transdiagnostic theories of psychological therapy, where treatment of a primary diagnosis such as depressive symptoms can also improve symptoms of a comorbid condition due to shared psychological processes.^S56^ Future research should consider treatment duration as a moderator, while also investigating possible differences in anxiety‐focused interventions.

Considering glycaemic control as a physiological measure of well‐being in diabetes, findings for CBT‐based interventions appear mixed. While some reviews showed improvements in HbA1c levels post‐intervention, with further improvements up to 8 months later, most reviews suggested no changes post‐intervention. There are several factors that make it difficult to draw conclusions from this literature. Reviews examining HbA1c as an outcome^4^, ^21^, ^23^, ^26^, ^42^ included studies examining HbA1c both as a primary or secondary outcome, but typically did not differentiate the intervention focus in their analysis. As also seen for anxiety, this may present a confounding factor, as targeting an intervention specifically can lead to better outcomes.^S57^ Despite finding no effectiveness immediately post‐intervention, Dong et al.^26^ suggested a moderate reduction in HbA1c levels over 6 months after treatment. This long‐term improvement may be explained by an indirect association between depressive symptoms and HbA1c levels.^S58^ An improvement in depressive symptoms is suggested to increase self‐management, subsequently improving HbA1c. Methodological challenges may confound findings, as HbA1c levels indicate blood glucose levels of the previous 3 months.^42^ Depending on treatment duration, measurements taken at the end of treatment may include HbA1c levels when improvements had not yet occurred. While most reviews do not examine long‐term glycaemic control, the reviews that do^20^, ^25^ evidence no statistical heterogeneity, suggesting a lasting improvement in HbA1c levels across time. Further research that addresses methodological issues would be helpful to improve confidence in these findings and inform clinical practice.^S58^

### 4.1 CBT therapy delivered through specialist practice

In comparison to CBT‐based interventions, CBT delivered by CBT therapists seems to be more effective in reducing DRD, and findings were maintained 9 months post‐intervention. This is supported by Jenkinson et al.,^20^ who suggested CBT effectiveness for DRD to be moderated by the delivery of a trained psychologist. CBT therapy appears to be equally effective in reducing depressive symptoms as CBT‐based interventions. This similarity was also shown for long‐term depressive symptoms, as gains were maintained but slightly reduced.^44^, ^48^ Non‐significant findings may be accounted for by mild baseline symptoms^47^, ^49^ or lack of a suitable control group at follow‐up.^46^ In contrast to review findings, anxiety symptoms were largely improved and maintained at 9 months, following 6–8 individual CBT sessions delivered by a CBT therapist.^42^, ^45^ This could be attributable to specialist practice or be related to the tendency for anxiety symptoms not to be considered a primary outcome in reviews.

Examining glycaemic control, CBT delivered in line with specialist practice demonstrates similar findings as seen within reviews. Most studies showed no improvements at the end of treatment, but improvements were found after 6 months,^48^ supporting the indirect link between depressive symptoms and HbA1c levels in depression‐targeted interventions. Further, Lutes et al.^43^ demonstrated an improvement in HbA1c levels immediately post‐intervention, supporting previous suggestions of a direct biological link between DRD and blood glucose levels in Type 2 diabetes.^S56^

Despite the promising findings of CBT effectiveness across outcomes for Type 1 and Type 2 diabetes, the quality of the included reviews varies. Two reviews were appraised as ‘low’ quality^40^, ^42^ introducing a risk of bias, but not excluded due to a limited number of reviews available. Five reviews did not reference risk of bias when interpreting results, potentially leading to a misestimation of treatment effects within reviews.^S60^ Six reviews only included published RCTs, suggesting an overestimation of effects may have occurred as positive findings have a greater likelihood of being published.^S61^ While all meta‐analyses utilised appropriate methodology to pool results, significant statistical heterogeneity across most reviews and a lack of sub‐analyses suggest caution when interpreting these findings for the effectiveness of CBT and CBT‐based interventions.

### 4.2 Strengths and limitations

The distinction of findings from RCTs conducted in line with specialist practice is a novel contribution of this umbrella review. Addressing the variation in previous reviews allows for greater confidence in interpreting these findings and making recommendations for future practice. However, the content of this umbrella review was restricted by the coverage and methods of existing systematic reviews and meta‐analyses. Considering differing aetiology of Type 1 and Type 2 diabetes,^2^ a focus on the more prevalent Type 2 diabetes may have been useful. This was not possible as most reviews did not analyse Type 2 diabetes separately. Furthermore, reviews did not consider whether the targeted focus in CBT (primary or secondary outcomes) impacted on findings. Reviews also included a variety of CBT interventions which were not differentiated within their analyses. While interventions conducted through specialist practice were distinguished here, further distinctions of other psychological practices would have exceeded the scope of this review. Validity and quality of this umbrella review is determined by the quality of the included reviews but also their included primary studies,^S62^ with potential inaccuracies in quality appraisal or information extraction, as well as any overlaps between included studies. As such, 33 of a total of 194 underlying studies were included in more than one review (17.01%). Further, only published systematic reviews and meta‐analyses were included, possibly inferring publication bias, while the language restriction (English language only) limits generalisability.

Taking methodological limitations into account, the findings of this umbrella review provide important additions to previous clinical guidance.^18^ The findings support previous recommendations in clinical guidance that both CBT and low‐intensity CBT‐based interventions can improve DRD and depressive symptoms. While the Matrix^18^ only recommended individual high‐intensity CBT for glycaemic control, current findings indicate that CBT‐based interventions may also be of benefit. This review adds to evidence that depression‐targeted individual CBT can lead to improvements in anxiety symptoms. The findings indicate that specialist CBT practice has a greater benefit for DRD and anxiety symptoms, and this may be helpful in considering who to refer for specialist therapy. The similar effectiveness outcomes in depressive symptoms and HbA1c levels for enhanced and skilled CBT practice may benefit service through greater options for staff to deliver the interventions and due to the potential for group‐based CBT‐based interventions to be cost‐effective and promote interpersonal support among people with diabetes.^S63^

Future research should aim to reduce clinical and methodological variability, as significant heterogeneity was established for all included reviews. This includes more investigation in differences between Type 1 and Type 2 diabetes as well as consistency in the psychometric tools used to assess outcome. As the current umbrella review distinguished CBT conducted through specialist practice, future research may benefit from further distinctions of enhanced or skilled practice. Previous literature suggests improvements in common mental health disorders differ according to CBT practice, with highest benefits seen for specialist practice with high treatment duration.^S64^ As CBT content differed widely, investigating possible moderators such as treatment duration may also reduce clinical variance.

## 5 CONCLUSION

This umbrella review suggests CBT‐based interventions provided by multidisciplinary staff are effective at reducing depressive symptoms, diabetes‐related distress and long‐term HbA1c levels. CBT‐based interventions did not reduce anxiety symptoms overall, although treatment duration may moderate this relationship. In comparison, CBT interventions delivered by a CBT therapist were more effective at reducing diabetes‐related distress and anxiety symptoms as a secondary measure, whereas improvements in depressive symptoms and glycaemic control were similar across CBT practice types. Given methodological limitations and statistical heterogeneity across reviews, further research is needed to confirm these results.

## FUNDING INFORMATION

The authors received no specific funding for this work.

## CONFLICT OF INTEREST STATEMENT

The authors report there are no competing interests to declare.

## Supporting information


Data S1.

Data S2.

## Data Availability

The authors confirm that the data supporting the findings of this study are available within the article [and/or] its supplementary materials.
