# Supplementary material for: How effective are CBT and CBT‐based interventions in Type 1 and Type 2 diabetes? An umbrella review
Source: Diabet Med. 2026 Feb 20;43(5):e70271. doi: 10.1111/dme.70271 (PMC13074150; doi:10.1111/dme.70271)
Supplement: Supplementary file 1 — Data S1. [file DME-43-e70271-s001.zip › dme70271-sup-0003-Supinfo1.docx]

Doc. S1. Supplementary references

S51. Menting J, Tack CJ, van Bon AC, et al. Web-based cognitive behavioural therapy blended with face-to-face sessions for chronic fatigue in type 1 diabetes: a multicentre randomised controlled trial. *The Lancet: Diabetes & Endocrinology*. 2017;5(6):448-456. doi:https://doi.org/10.1016/s2213-8587(17)30098-0

S52. Rizzo M, Creed F, Goldberg D, Meader N, Pilling S. A systematic review of non-pharmacological treatments for depression in people with chronic physical health problems. *Journal of Psychosomatic Research*. 2011;71(1):18-27. doi:https://doi.org/10.1016/j.jpsychores.2011.02.011

S53. Melsen WG, Bootsma MCJ, Rovers MM, Bonten MJM. The effects of clinical and statistical heterogeneity on the predictive values of results from meta-analyses. *Clinical Microbiology and Infection*. 2014;20(2):123-129. doi:https://doi.org/10.1111/1469-0691.12494

S54. Shapiro DA, Barkham M, Rees A, Hardy GE, Reynolds S, Startup M. Effects of treatment duration and severity of depression on the effectiveness of cognitive-behavioral and psychodynamic-interpersonal psychotherapy. *Journal of Consulting and Clinical Psychology*. 1994;62(3):522-534. doi:https://doi.org/10.1037/0022-006x.62.3.522

S55. Nefs G, Pouwer F, Denollet J, Pop V. The course of depressive symptoms in primary care patients with type 2 diabetes: results from the Diabetes, Depression, Type D Personality Zuidoost-Brabant (DiaDDZoB) Study. *Diabetologia*. 2011;55(3):608-616. doi:https://doi.org/10.1007/s00125-011-2411-2

S56. Brown TA, Barlow DH. Comorbidity among anxiety disorders: Implications for treatment and DSM-IV. *Journal of Consulting and Clinical Psychology*. 1992;60(6):835-844. doi:https://doi.org/10.1037/0022-006x.60.6.835

S57. Craske MG. The future of CBT and evidence‐based psychotherapies is promising. *World Psychiatry*. 2022;21(3):417-419. doi:https://doi.org/10.1002/wps.21002

‌

S58. Schmitt A, Bendig E, Baumeister H, Hermanns N, Kulzer B. Associations of depression and diabetes distress with self-management behavior and glycemic control. *Health Psychology*. 2020;40(2). doi:https://doi.org/10.1037/hea0001037

S59. Sandercock P. The Authors Say: “The Data Are Not So Robust because of Heterogeneity” – So, How Should I Deal with This Systematic Review. *Cerebrovascular Diseases*. 2011;31(6):615-620. doi:https://doi.org/10.1159/000326068

S60. Higgins JPT, Altman DG, Gotzsche PC, et al. The Cochrane Collaboration’s Tool for Assessing Risk of Bias in Randomised Trials. *BMJ*. 2011;343. doi:https://doi.org/10.1136/bmj.d5928

S61. Mlinarić A, Horvat M, Šupak Smolčić V. Dealing with the positive publication bias: Why you should really publish your negative results. *Biochemia Medica*. 2017;27(3). doi:https://doi.org/10.11613/bm.2017.030201

S62. Belbasis L, Bellou V, Ioannidis JPA. Conducting umbrella reviews. *BMJ Medicine*. 2022;1(1). doi:https://doi.org/10.1136/bmjmed-2021-000071

S63. Due-Christensen M, Zoffmann V, Hommel E, Lau M. Can sharing experiences in groups reduce the burden of living with diabetes, regardless of glycaemic control? *Diabetic Medicine*. 2012;29(2):251-256. doi:https://doi.org/10.1111/j.1464-5491.2011.03521.x

S64. Robinson L, Kellett S, Delgadillo J. Dose‐response patterns in low and high intensity cognitive behavioral therapy for common mental health problems. *Depression and Anxiety*. 2020;37(3). doi:https://doi.org/10.1002/da.22999
