# Supplementary material for: How effective are CBT and CBT‐based interventions in Type 1 and Type 2 diabetes? An umbrella review
Source: Diabet Med. 2026 Feb 20;43(5):e70271. doi: 10.1111/dme.70271 (PMC13074150; doi:10.1111/dme.70271)
Supplement: Supplementary file 1 — Data S1. [file DME-43-e70271-s001.zip › dme70271-sup-0004-Supinfo2.docx]

**Appendices and Supporting Information (supplied as separate files)**

**Appendix S1 :** Supplementary Tables (Tables longer than 2 pages)

**Table S1.** *Characteristics of included reviews*

| **Review, Year,**  **Country,**  **Short Title** | **Review type**  **Quality appraisal tool used** | **Study:**  **Design**  **Number**  **Country of CBT studies** | **Sample:**  **Total participants**  **Age range**  **% female**  **Diagnosis** | **Interventions**  **Controls** | **CBT Intervention moderators** | **Overview of review outcomes following CBT** |
| --- | --- | --- | --- | --- | --- | --- |
| An et al. (2023)[^19^](#Reference19)  China/USA  The effectiveness of cognitive behavioral therapy for depression among individuals with diabetes. | Systematic review and meta-analysis  Cochrane risk-of-bias tool for randomized trials (RoB 2) | Controlled trial design (RCTs or controlled trial without random assignment)  RCTs  Total: 33  CBT: 33  North America (12), Europe (12), China (2), Australia (3), Egypt (2), Iran (2) | 4268  Mean age 55.12 years  62.23% females (n=2813)  T1DM (11)  T2DM (19)  T1DM and T2DM (1)  Not specified (1)  All participants had comorbid depression. | CBT intervention for depressive symptoms (studies must detail CBT model underlying intervention, if CBT informed must have at least 2: cognitive restructuring, behavioural activation, problem solving. Problem solving therapy considered CBT. Third-wave CBT excluded).  Control: N/R | Outcome-related effects: CBT effectiveness greater for depressive symptoms than physiological outcomes. | For DRD a small treatment effect was found.  Meta-analyses found CBT to have a moderate effect on depressive symptoms. Findings for anxiety symptoms showed a non-significant treatment effect.  Physiological outcomes  (HbA1c, fasting glycaemia, cholesterol level) non-significantly improved. |
| Dong et al. (2023)[^26^](#Reference26)  China  The short- and long-term effects of cognitive behavioral therapy on the glycemic control of diabetic patients. | Systematic review and meta-analysis  Cochrane risk of bias tool | RCTs  Total: 19  CBT: 19  USA (6), Sweden (1), Australia (3), Netherlands (4), Germany (1), UK (1), China (3) | N/R  Mean ages across studies between 38.1 – 63.9 years. 2 adolescent studies mean age of 13.2 to 15.2 years.  N/R  T1DM (7)  T2DM (10)  Both (2) | ‘Pure’ CBT treatment, not mixed with other interventions, delivered in a range of formats (group, individual, web-based), no details provided on individuals delivering CBT, targeting various outcomes, must include glycaemic control measures.  Control: Usual care | N/A | HbA1c levels measured short-term (< 6 months) after CBT were non-significant. If HbA1c levels were measured long-term (> 6 months) treatment effects were significant. A subgroup analysis with diabetes type showed this to be significant in both T1DM and T2DM. |
| Fiqri et al. (2022)[^24^](#Reference24)  Indonesia  Cognitive Behavioral Therapy for self-care behaviors with type 2 diabetes mellitus patients. | Systematic review  Critical Appraisal Skill Program, Cochrane risk of bias, GRADE | RCTs  Total: 7  CBT: 7  USA (4), Netherlands (1), China (1), Australia (1) | 368  Mean ages across studies 52,60 – 63,42 years  N/R  T2DM (5 studies comorbid depressive symptoms) | CBT programmes for self-care behaviours (Delivered by psychologists and multidisciplinary staff)  Control: non-CBT interventions, CBT combination, usual care, waitlist | Duration of treatment had sig. effect at 3-12 months, between 12-21 sessions. Group CBT sig. effect on overall self-care behaviours, individual CBT only some components. | DRD showed sig effect in 2 studies, and non-sig. effect in 1 study.  CBT was found to be effective on depressive symptoms in 3 studies, a non-sig. effect was found in 1 study. No significant effect on anxiety symptoms (*n* = 1).  Mixed findings for HbA1c in short and long-term (*n* = 4).  Sig. improved self-care behaviours. |
| Jenkinson et al. (2022)[^20^](#Reference20)  UK  The effectiveness of Cognitive Behavioural Therapy and third-wave Cognitive Behavioural interventions on diabetes‐related distress. | Systematic review and meta-analysis  Cochrane Risk of Bias Tool | RCTs, ‘quasi’-RCTs  Total: 22  CBT: 18  Sweden (1), Malaysia (1), Australia (2), USA (4), Germany (2), UK (1), Norway (1), Netherlands (4), Croatia (1), Thailand (1) | 4123  Mean age 37.8 – 70.7 years  54.9% female  T2DM (10, CBT:8)  T1DM (2, CBT:2)  Both (10, CBT:7) | CBT targeting diabetes-related distress (cognitive and behavioural component, delivered in a range of formats and professionals).  Third-wave CBT (ACT/mindfulness-based interventions [MBI]).  Control: usual care, waitlist, placebo, active control. | Delivery by psycho-logical therapist, individual format, including behavioural activation and digital components moderated improvements on DRD. | Small effect of CBT for DRD on DRD. Slightly larger effect size if DRD is primary outcome than if also measured as secondary outcome.  Moderate effect on depressive symptoms (*n* = 3), 1 study for anxiety symptoms evidenced a large effect.  Small non-sig. effect on glycaemic control (HbA1c levels). |
| Mather et al. (2022)[^41^](#Reference41)  UK  The limited efficacy of psychological interventions for depression in people with Type 1 or Type 2 diabetes. | Systematic review and individual Participant data meta-analysis  Cochrane Risk of Bias Tool-2 (RoB2) | RCTs  Total: 25 (12 with IPD)  CBT: 8 providing IPD, 7 no IPD.  Only IPD data: Netherlands (3), Germany (1), USA (1), Australia (1), Croatia (1), UK (1) | 2106 (IPD included 1385 due to missing data)  Median age 56 (range 18 to 91)  64.3% female  74.1% had T2DM (no further detail provided) | Pharmacological interventions (no IPD).  CBT targeting depression (no definition of what this entails).  Mindfulness-based interventions (*n* = 2), ACT (*n* = 1), education (*n* = 1), Therapist-Assisted (*n* = 1)  Control: usual care, waitlist, active control. | Intervention (MBI over CBT) and outcome measure (CESD over BDI or PHQ-9) moderated recovery and improvement in depressive symptoms. | Depressive individuals more likely to recover with MBI than CBT. Low CBT recovery rate (17%). Also, low CBT improvement rate (risk difference = 0.20).  Overall finding were low recovery rates of individuals with depressive symptoms across the investigated psychological interventions. |
| Oyedeji et al. (2022)[^42^](#Reference42)  UK  Effectiveness of non-specialist delivered psychological interventions on glycemic control and mental health problems in individuals with type 2 diabetes. | Systematic review and meta-analysis  Cochrane Risk of Bias tool | RCTs  Total: 16 (11 in meta-analysis)  CBT: 6  USA (3), Netherlands (2), Taiwan (1) | 4863  Mean age between 50-70.7 years  More female than male participants in 8 studies, 1 study only females.  T2DM | CBT delivered by non-specialists, range of formats, targeting glycaemic control, depression, DRD.  Other: Motivational Interviewing (MI, *n* = 8), combination of CBT and MI (*n* = 1), MCBT (*n* ­= 1)  Control: usual care, diabetes education, waitlist | Delivery by non-specialist multidisciplinary staff. | Effect size not calculated for DRD or depressive symptoms due to various scales. CBT studies did not improve DRD. For depressive symptoms, CBT showed mixed findings, with some improvements (not maintained at 12 months), others showed non-significant findings.  Non-significant effect for HbA1c levels following CBT. |
| Winkley et al. (2020)[^4^](#Reference4)  UK  Psychological interventions to improve self-management of type 1 and type 2 diabetes. | Systematic review and meta-analysis, network meta-analysis, IPD meta-analysis, cost-effectiveness modelling  Cochrane Risk of Bias for randomised and non-randomised controlled trials | RCTs (non-RCTs and adolescent studies not detailed here)  Total: 96  Type 1: 18, of these CBT (5):  Europe (5)  Type 2 adults: 71, of these CBT (19):  Europe (7), North America (6), Asia (5), Australia (1) | 18,659  Mean ages across CBT studies: 34.2 to 57 years.  N/R  Adults Type 1 (1457 participants)  Adults Type 2 (14,326 participants) | CBT targeting self-management (HbA1c) (Delivered by psychologists and multidisciplinary staff, range of formats).  Other interventions: counselling, collaborative care, creative therapy, family therapy (number of studies not provided).  Control: usual care, diabetes education, waitlist | N/A | Adults T1DM: non-significant HbA1c decrease for psychological interventions and no differences between interventions.  Adults T2DM: Sub analysis showed significant reduction of HbA1c following CBT.  Psychological interventions overall (CBT not differentiated) showed a significant decrease in depressive symptoms. |
| Li et al. (2017)[^22^](#Reference22)  China  A systematic review and meta-analysis of randomized controlled trials of cognitive behavior therapy for patients with diabetes and depression. | Systematic review and meta-analysis  Cochrane Risk of Bias tool | RCTs  Total: 10  CBT: 10  China (2), Iran (1), Germany (1), Netherlands (1), USA (4), Taiwan (1) | 998  Mean ages across studies between 43.26-58.31 years  N/R  T2DM and comorbid depression (8)  Both with comorbid depression (2) | CBT or CBT-based interventions targeting depressive symptoms, duration detailed as short-term or long-term (no further definition of what intervention entails).  Control: usual care, diabetes education, Citalopram hydrobromide | Intervention length: short-term (2-6 months) or long-term (6-12 months). Both were effective for depressive symptoms, only long-term CBT effective for anxiety symptoms. | Non-significant effect size of CBT for DRD or HbA1c levels.  Moderate effect on depressive symptoms, sub-analyses showed large effect size when short-term duration, moderate for long-term duration.  Overall non-significant effect on anxiety symptoms but sub-analyses showed a small effect size if long-term CBT. |
| Uchendu & Blake (2017)[^21^](#Reference21)  UK  Effectiveness of cognitive-behavioural therapy on glycaemic control and psychological outcomes in adults with diabetes mellitus. | Systematic review and meta-analysis  Cochrane Risk of Bias tool | RCTs  Total: 12 (9 in meta-analysis)  CBT: 12  USA (5), Netherlands (4), Australia (1), Sweden (1), Iran (1) | 1445  Mean ages between 37.4 +/- 11.1 and 61 +/- 10.8 years  N/R  T1DM (3)  T2DM (7)  Both (2)  All participants had comorbid depressive symptoms. | CBT: cognitive and behavioural components, more than one session, delivered in a range of formats (group/individual) and health professionals with different degrees of training in CBT. Primary outcome glycaemic control, secondary outcomes includes DRD, anxiety, depression  Control: treatment as usual, non-CBT interventions, waitlist | N/A | Improvement in DRD up to 4 months, mixed results medium and long-term, no meta-analysis due to varying measures.  Large effect size in short-term, moderate in medium-term depressive symptoms, small in long-term.  Moderate effect size for short-term and medium-term anxiety symptoms. Large effect size for long-term anxiety symptoms but based on 1 study.  HbA1c levels significantly improved in short-term, even more in medium-term. Effect was not sustained up to 12 months. |
| Wang et al. (2017)[^40^](#Reference40)  China  Cognitive behavioural therapy on improving the depression symptoms in patients with diabetes. | Meta-analysis  Cochrane Collaboration tool for Risk of Bias | RCTs  Total: 5  CBT: 5  Netherlands (2), Germany (1), USA (2) | 834  Mean ages across studies 37.8 to 61.2 years  50.5% female  T1DM (1)  T2DM (3)  Both (1) | CBT intervention targeting depression (no definition of what this entails).  Control: usual care, other non-CBT interventions | N/A | For depressive symptoms a moderate treatment effect of CBT was found post-treatment. This was maintained at 12 months.  When the Centre for Epidemiological Studies Scale for Depression (CES-D) was used to assess effectiveness of CBT on depressive symptoms, findings were non-significant. |
| Chapman et al. (2015)[^23^](#Reference23)  Australia  Psychological interventions for the management of glycemic and psychological outcomes of type 2 diabetes mellitus in China. | Systematic review and meta-analysis  Cochrane Risk of Bias tool | RCTs  Total: 48 (45 in meta-analysis)  CBT: 12 (associated techniques e.g., relaxation training k=25), (20 in meta-analysis)  N/R | N/R, sample size ranged from 59 to 598  Mean age 52.8 +/- 11.8 years  N/R  T2DM (35)  T2DM with comorbid depressive symptoms/anxiety symptoms (10) | CBT (behavioural and cognitive components, delivered in a range of formats, by psychologists and multidisciplinary staff). Primary outcome glycated haemoglobin, secondary anxiety and depression symptoms.  MI, client-centred therapy, psychodynamic psychotherapy, interpersonal psychotherapy  Control: usual care, waitlist, education. | N/A | Large effect size of CBT for depressive symptoms and for anxiety symptoms.  Large significant effect of CBT on glycaemic control (HbA1c levels). |

**Table S2.** *Characteristics of included studies*

| **Study author**  **Year**  **Country**  **Nr of reviews found within** | **Design**  **Statistical methods** | **Sample:**  **Sample size**  **Mean age**  **% female**  **Diagnosis and average duration** | **Intervention** | **Validated outcome measures** | **Overview of relevant study outcomes** |
| --- | --- | --- | --- | --- | --- |
| Higgins et al. (2022)[^48^](#Reference48)  USA  Found in 1 review: An et al. (2023)[^19^](#Reference19) | RCT  Mixed model for repeated measurements, t-tests. | 47 (23 CBT, 24 control)  62.3 ± 8.42  6.4% female  T2DM with peripheral neuropathic pain; median duration 120 months | CBT for chronic pain with modifications for diabetic peripheral neuropathic pain  **Format:** 10 individual 60-min sessions across 14 weeks face to face.  **Delivered by:** psychologists at doctorate level with supervision.  **Content:** relaxation, psychoeducation, sleep hygiene, behavioural activation, activity pacing, cognitive restructuring, diabetes self-care, relapse prevention  **Control:** diabetes education | Depressive symptoms: BDI  Pain-relevant measures (detailed in study) | Non-significant change for depressive symptoms (*p* = 0.26). Note mild symptoms at baseline. |
| Alshehri et al. (2021)[^46^](#Reference46)  USA  Found in 2 reviews: An et al. (2023)[^19^](#Reference19), Dong et al. (2023)[^26^](#Reference26) | Pilot RCT  Independent samples t-tests and Mann Whitney U tests. | 28 (13 in each group)  61.86 ± 6.48 in CBT-I group  71.42% female  T2DM; average duration 15.71 years ± 9.93 in CBT-I group | CBT for Insomnia (CBT-I)  **Format:** Six face to face weekly individual 60-minute sessions.  **Delivered by:** trained CBT-I providers with supervision.  **Content:** cognitive therapy, sleep restriction, stimulus control, sleep management, relaxation techniques.  **Control:** Health education group | Depressive symptoms: BDI  Anxiety symptoms: GAD-7 | Large improvements in depressive symptoms following CBT (Cohen’s *d* = 1.49, *p* = .002).  Large improvements in anxiety symptoms following CBT (Cohen’s *d* = 0.88, *p* = .04). |
| Lutes et al. (2020)[^44^](#Reference44)  USA  Found in 2 reviews: An et al. (2023)[^19^](#Reference19), Fiqri et al. (2022)[^24^](#Reference24) | RCT  t-tests to compare groups. | 139  Non-insulin users (53.35 ± 8.69), Insulin users (51.90 ± 6.31)  Non-insulin users (76.1% female), Insulin users (79.2% female)  T2DM and comorbid depressive or regimen-related distress; average duration 11.15 years ± 8.95. | CBT tailored to symptom severity  **Format:** 16 face to face sessions over 12 months either CBT or small changes lifestyle coaching. (Findings not differentiated, 72% received CBT, 28% received coaching.)  **Delivered by:** clinical health psychologist and/or doctorate student in clinical health psychology.  **Content:** challenging diabetes-related cognitive distortions, behavioural techniques (e.g., behavioural activation), adaptive coping strategies from problem-solving interventions.  **Control:** usual care | DDS-17 (Regimen-related distress subscore)  PHQ-9  HbA1c  Summary of Diabetes Self-Care Activities,  Medication Adherence Scale | Significant improvement regimen-related distress following CBT compared to control (*t* = 2.02, *p* = 0.05). In insulin users moderate positive correlation with HbA1c levels (*r* = 0.27, *p* = 0.05).  Non-significant improvement for depressive symptoms (*p* = 0.12).  Small improvements in HbA1c for insulin users (*t* = 1.85, *p* = 0.07). |
| De Groot et al. (2019)[^27^](#Reference27)  USA  Found in 3 reviews: An et al. (2023)[^19^](#Reference19), Jenkinson et al. (2022)[^20^](#Reference20), Mather et al., (2020)[^41^](#Reference41) | 2x2 factorial RCT  ANCOVA | 140  56.0 years ± 10.7  76.5% female  T2DM for less than 1 year and comorbid major depression; N/R | CBT for depression  **Format:** 10 individual sessions across 12 weeks face to face.  **Delivered by:** CBT trained community mental health providers (masters or doctorate level).  **Content:** based on Beck’s model of cognitive therapy, restructuring negative automatic thoughts, behavioural challenging.  **Other interventions:** EXER (community-based exercise), CBT + EXER or usual care | Depressive symptoms: BDI-II, Automatic Thoughts Questionnaire  Diabetes Distress Scale 17  HbA1c | Significant improvements in depression (*p* = 0.011) and DRD (*p* = 0.003). Remission rate 66%.  Non-significant differences of CBT and usual care for HbA1c levels (*p* = 0.379). |
| Menting et al. (2017)^S51^  Netherlands  Found in 1 review: Dong et al. (2023)[^26^](#Reference26) | RCT  Independent sample t-test and Chi-square tests, ANCOVA | 120  CBT group: 44.4 ± 12.1 years  62% female in CBT group  T1DM, average duration 24.2 ± 13.3 years. | CBT for chronic fatigue (Dia-Fit)  **Format:** 5-8 face to face 50 minute sessions, followed by 8 web-based sessions across 5 months.  **Delivered by:** clinical psychologists with supervision.  **Content:** behavioural and cognitive techniques, achieving goals, challenging negative beliefs.  **Control:** waitlist. | HbA1c | Following CBT, Non-significant change for HbA1c levels (*p* = 0.889). |
| Tovote et al. (2014)[^43^](#Reference43)  Netherlands  Found in 4 reviews: An et al. (2023)[^19^](#Reference19), Jenkinson et al. (2022)[^20^](#Reference20), Mather et al. (2020)[^41^](#Reference41), Li et al. (2017)[^22^](#Reference22) | RCT  ANOVA and Chi-Square tests. ANCOVAs for CBT and MCBT with waitlist control. | 94  53.1 ± 11.8  49% female  T1DM (39%) and T2DM (61%), average duration 16.6 ± 11.9 years. | CBT and Mindfulness-based CBT (analysed separately) aimed at reducing depression  **Format:** individual face to face 8 weekly sessions of 45–60-minute duration.  **Delivered by:** trained therapists receiving supervision.  **Content of CBT:** cognitive restructuring, behavioural activation.  **Control:** waitlist. | BDI-II  Toronto Hamilton Depression Rating Scale  GAD-7  Wel-Being Index  PAID  HbA1c | Large effect of CBT for depressive symptoms (*d* = 1.00 and 1.09, *p* < 0.001) for the BDI-II and HAM-D7 respectively.  Large effect of CBT for anxiety symptoms (*d* = 0.82, *p* = 0.01), well-being measure (*d* = 1.02, *p* < 0.001). Moderate effect for DRD (*d* = 0.57, *p* = 0.04).  Non-significant reduction for HbA1c levels following CBT (*p* = 0.72). |
| Tovote et al. (2015)[^45^](#Reference45)  Netherlands  Found in 1 review: An et al. (2023)[^19^](#Reference19) | Study design, sample, measures, and intervention are the same as seen for Tovote et al. (2014), this study differs only in analysed outcomes. While Tovote et al. (2014) investigates immediate post-intervention outcomes, this study looked at long-term follow-up at 9 months. | | | | At 9-month follow-up moderate effect on depressive symptoms (*d* = 0.62, *p* < 0.001), moderate to large effect on anxiety symptoms (*d* = 0.79, *p* < 0.001), and moderate effect on diabetes-related distress (*d* = 0.35, *p* = 0.002).  Non-significant change in HbA1c levels at 9 months follow-up (*p* = 0.38). (Note: effect size calculated from pre-treatment to 9-month follow-up.) |
| Safren et al. (2014)[^47^](#Reference47)  USA  Found in 7 reviews: An et al. (2023)[^19^](#Reference19), Dong et al. (2023)[^26^](#Reference26), Fiqri et al. (2022)[^24^](#Reference24), Li et al. (2017)[^22^](#Reference22), Uchendu & Blake (2017)[^21^](#Reference21), Wang et al. (2017)[^40^](#Reference40) Winkley et al. (2020)[^4^](#Reference4) | RCT  Mean differences calculated. | 87  56.88 years  49.43% female  Uncontrolled T2DM, and comorbid depressive symptoms, duration not reported. | CBT-Adherence  **Format:** Initial session diabetes-self-management education. Then 9-12 CBT sessions, primarily in person.  **Delivered by:** Initial Life-steps session is delivered by a nurse and dietician, rest of the sessions conducted by a therapist (unclear about exact training).  **Content:** Behavioural activation, cognitive restructuring, problem-solving and relaxation training.  **Control:** Treatment as usual. | Montgomery-Asberg Depression Rating Scale  Clinical Global Impression  HbA1c  Adherence to glucose monitoring | Reduced depressive symptoms post-intervention (6.44 lower scores on MADRS, *p* = 0.002 and 0.74 lower scores on CGI, *p* = 0.01). At follow-up of 8 and 12 months, there was a movement towards further improvement, but findings were non-significant (*p* = 0.16).  Improved HbA1c levels following CBT treatment, (0.72 difference, *p* = 0.001). This was maintained at 8 months (*p* = 0.03). |
| Otis et al. (2013)[^50^](#Reference50)  USA  Found in 1 review: An et al. (2023)[^19^](#Reference19) | RCT pilot study  ANOVAs, hierarchical linear models, paired-sample t-tests | 20  62.94 years  N/R  T2DM | CBT for diabetic peripheral neuropathy  **Format:** face to face, 11x 60-minute individual CBT pain management sessions.  **Delivered by:** Clinical psychologist (doctorate level), or therapist (master level), supervision provided.  **Content:** pain management, relaxation techniques, challenging maladaptive thoughts, sleep, anger.  **Control:** Treatment as usual. | BDI | Non-significant change in depressive symptoms pre-treatment to the 4 month follow-up (*p* not provided). |
| Lustman et al. (1998)[^49^](#Reference49)  USA  Found in 5 reviews: An et al. (2023)[^19^](#Reference19), Dong et al. (2023)[^26^](#Reference26), Mather et al. (2022)[^41^](#Reference41), Li et al. (2017)[^22^](#Reference22), Uchendu & Blake (2017)[^21^](#Reference21) | RCT  ANOVA and ANCOVA | 51  CBT group: 53.1 ± 10.5 years  60% females in CBT group  T2DM and comorbid depression, average duration in CBT group 9.9 ± 11.8 years. | CBT  **Format:** 60-minute weekly face to face individual sessions, and every 2 weeks a 60-minute diabetes education programme. This was completed across 10 weeks.  **Delivered by:** certified CBT therapist/psychologist, and a diabetes educator for education sessions.  **Content:** problem-solving strategies, behavioural strategies (behavioural activation), cognitive restructuring.  **Control:** antidepressant treatment with diabetes education programme. | Glycaemic control was measured with GHb levels  BDI | Non-significant differences between CBT and control group for glycaemic control (*p* = 0.17). However, at 6-month follow-up GHb levels significantly improved in the CBT group (*p* = 0.04).  Following CBT, individuals displayed an 85% remission rate in depressive symptoms symptoms (*p* < 0.001). At 6-month follow-up remission rate was 70% for depressive symptoms (*p* = 0.03). |

**Appendix S2 :** Detailed quality appraisal

| **Authors, date** | **(1) PICO** | **(2) Search** | **(3) Study selec-tion** | **(4) Extrac-tion** | **(5) publication status** | **(6) Exclusion** | **(7) Included studies** | **(8)**  **quality assessment** | **(9)**  **quality discussion** | **(10) Combi-nation** | **(11) Publication bias** | **(12) Conflict of interest** | **Overall rating** |
| --- | --- | --- | --- | --- | --- | --- | --- | --- | --- | --- | --- | --- | --- |
| An et al. (2023)[^19^](#Reference19) | Y | Y | Y | Y | Y | N | Y | Y | N | Y | Y | Y | High |
| Dong et al. (2023)[^26^](#Reference26) | Y | Y | Y | Y | N | N | Y | Y | N | Y | N | Y | Acceptable |
| Fiqri et al. (2022)[^24^](#Reference24) | Y | Y | Y | Y | Y | N | Y | Y | N | N/A | N/A | Y | Low |
| Jenkinson et al. (2022)[^20^](#Reference20) | Y | Y | Y | Y | N | N | Y | Y | Y | Y | Y | Y | High |
| Mather et al. (2022)[^41^](#Reference41) | Y | Y | Y | Y | N | N | Y | Y | N | Y | N | Y | Acceptable |
| Oyedeji et al. (2022)[^42^](#Reference42) | Y | Y | Y | Y | N | N | Y | Y | Y | Y | Y | Y | Low |
| Winkley et al. (2020)[^4^](#Reference4) | Y | Y | Y | Y | Y | N | Y | Y | Y | Y | Y | Y | High |
| Li et al. (2017)[^22^](#Reference22) | Y | Y | Y | Y | Y | N | Y | Y | Y | Y | N | Y | Acceptable |
| Uchendu & Blake (2017)[^21^](#Reference21) | Y | Y | Y | Y | Y | N | Y | Y | Y | Y | Y | Y | Acceptable |
| Wang et al. (2017)[^40^](#Reference40) | Y | Y | Y | C | N | N | Y | Y | N | Y | Y | Y | Low |
| Chapman et al. (2015)[^23^](#Reference23) | Y | Y | Y | Y | N | N | Y | Y | Y | Y | Y | Y | High |

**Appendix S3 :** Supplementary references (s51-s64)

S51. Menting J, Tack CJ, van Bon AC, et al. Web-based cognitive behavioural therapy blended with face-to-face sessions for chronic fatigue in type 1 diabetes: a multicentre randomised controlled trial. *The Lancet: Diabetes & Endocrinology*. 2017;5(6):448-456. doi:https://doi.org/10.1016/s2213-8587(17)30098-0

S52. Rizzo M, Creed F, Goldberg D, Meader N, Pilling S. A systematic review of non-pharmacological treatments for depression in people with chronic physical health problems. *Journal of Psychosomatic Research*. 2011;71(1):18-27. doi:https://doi.org/10.1016/j.jpsychores.2011.02.011

S53. Melsen WG, Bootsma MCJ, Rovers MM, Bonten MJM. The effects of clinical and statistical heterogeneity on the predictive values of results from meta-analyses. *Clinical Microbiology and Infection*. 2014;20(2):123-129. doi:https://doi.org/10.1111/1469-0691.12494

S54. Shapiro DA, Barkham M, Rees A, Hardy GE, Reynolds S, Startup M. Effects of treatment duration and severity of depression on the effectiveness of cognitive-behavioral and psychodynamic-interpersonal psychotherapy. *Journal of Consulting and Clinical Psychology*. 1994;62(3):522-534. doi:https://doi.org/10.1037/0022-006x.62.3.522

S55. Nefs G, Pouwer F, Denollet J, Pop V. The course of depressive symptoms in primary care patients with type 2 diabetes: results from the Diabetes, Depression, Type D Personality Zuidoost-Brabant (DiaDDZoB) Study. *Diabetologia*. 2011;55(3):608-616. doi:https://doi.org/10.1007/s00125-011-2411-2

S56. Brown TA, Barlow DH. Comorbidity among anxiety disorders: Implications for treatment and DSM-IV. *Journal of Consulting and Clinical Psychology*. 1992;60(6):835-844. doi:https://doi.org/10.1037/0022-006x.60.6.835

S57. Craske MG. The future of CBT and evidence‐based psychotherapies is promising. *World Psychiatry*. 2022;21(3):417-419. doi:https://doi.org/10.1002/wps.21002

‌

S58. Schmitt A, Bendig E, Baumeister H, Hermanns N, Kulzer B. Associations of depression and diabetes distress with self-management behavior and glycemic control. *Health Psychology*. 2020;40(2). doi:https://doi.org/10.1037/hea0001037

S59. Sandercock P. The Authors Say: “The Data Are Not So Robust because of Heterogeneity” – So, How Should I Deal with This Systematic Review. *Cerebrovascular Diseases*. 2011;31(6):615-620. doi:https://doi.org/10.1159/000326068

S60. Higgins JPT, Altman DG, Gotzsche PC, et al. The Cochrane Collaboration’s Tool for Assessing Risk of Bias in Randomised Trials. *BMJ*. 2011;343. doi:https://doi.org/10.1136/bmj.d5928

S61. Mlinarić A, Horvat M, Šupak Smolčić V. Dealing with the positive publication bias: Why you should really publish your negative results. *Biochemia Medica*. 2017;27(3). doi:https://doi.org/10.11613/bm.2017.030201

S62. Belbasis L, Bellou V, Ioannidis JPA. Conducting umbrella reviews. *BMJ Medicine*. 2022;1(1). doi:https://doi.org/10.1136/bmjmed-2021-000071

S63. Due-Christensen M, Zoffmann V, Hommel E, Lau M. Can sharing experiences in groups reduce the burden of living with diabetes, regardless of glycaemic control? *Diabetic Medicine*. 2012;29(2):251-256. doi:https://doi.org/10.1111/j.1464-5491.2011.03521.x

S64. Robinson L, Kellett S, Delgadillo J. Dose‐response patterns in low and high intensity cognitive behavioral therapy for common mental health problems. *Depression and Anxiety*. 2020;37(3). doi:https://doi.org/10.1002/da.22999
