# Supplementary material for: How effective are CBT and CBT‐based interventions in Type 1 and Type 2 diabetes? An umbrella review
Source: Diabet Med. 2026 Feb 20;43(5):e70271. doi: 10.1111/dme.70271 (PMC13074150; doi:10.1111/dme.70271)
Supplement: Supplementary file 1 — Data S1. [file DME-43-e70271-s001.zip › dme70271-sup-0001-AppendixS2.docx]

**Appendix S2 :** Detailed quality appraisal

| **Authors, date** | **(1) PICO** | **(2) Search** | **(3) Study selec-tion** | **(4) Extrac-tion** | **(5) publication status** | **(6) Exclusion** | **(7) Included studies** | **(8)**  **quality assessment** | **(9)**  **quality discussion** | **(10) Combi-nation** | **(11) Publication bias** | **(12) Conflict of interest** | **Overall rating** |
| --- | --- | --- | --- | --- | --- | --- | --- | --- | --- | --- | --- | --- | --- |
| An et al. (2023)[^19^](#Reference19) | Y | Y | Y | Y | Y | N | Y | Y | N | Y | Y | Y | High |
| Dong et al. (2023)[^26^](#Reference26) | Y | Y | Y | Y | N | N | Y | Y | N | Y | N | Y | Acceptable |
| Fiqri et al. (2022)[^24^](#Reference24) | Y | Y | Y | Y | Y | N | Y | Y | N | N/A | N/A | Y | Low |
| Jenkinson et al. (2022)[^20^](#Reference20) | Y | Y | Y | Y | N | N | Y | Y | Y | Y | Y | Y | High |
| Mather et al. (2022)[^41^](#Reference41) | Y | Y | Y | Y | N | N | Y | Y | N | Y | N | Y | Acceptable |
| Oyedeji et al. (2022)[^42^](#Reference42) | Y | Y | Y | Y | N | N | Y | Y | Y | Y | Y | Y | Low |
| Winkley et al. (2020)[^4^](#Reference4) | Y | Y | Y | Y | Y | N | Y | Y | Y | Y | Y | Y | High |
| Li et al. (2017)[^22^](#Reference22) | Y | Y | Y | Y | Y | N | Y | Y | Y | Y | N | Y | Acceptable |
| Uchendu & Blake (2017)[^21^](#Reference21) | Y | Y | Y | Y | Y | N | Y | Y | Y | Y | Y | Y | Acceptable |
| Wang et al. (2017)[^40^](#Reference40) | Y | Y | Y | C | N | N | Y | Y | N | Y | Y | Y | Low |
| Chapman et al. (2015)[^23^](#Reference23) | Y | Y | Y | Y | N | N | Y | Y | Y | Y | Y | Y | High |
