# Supplementary material for: How effective are CBT and CBT‐based interventions in Type 1 and Type 2 diabetes? An umbrella review
Source: Diabet Med. 2026 Feb 20;43(5):e70271. doi: 10.1111/dme.70271 (PMC13074150; doi:10.1111/dme.70271)
Supplement: Supplementary file 2 — Data S2. [file DME-43-e70271-s002.docx]

**S4 Excluded reviews with reason excluded**

**CBT not analysed separately**

1. Chew, B. H., Vos, R. C., Metzendorf, M., Scholten, R. J., & Rutten, G. E. (2017). Psychological interventions for diabetes-related distress in adults with type 2 diabetes mellitus. *The Cochrane Database of Systematic Reviews, 9*, CD011469. <https://doi.org/10.1002/14651858.CD011469.pub2>
2. Lu, X., Yang, D., Liang, J., Xie, G., Li, X., Xu, C., Liao, H., Zhou, H., Xu, Z., Ye, C., Chen, H., Liang, M., Shen, Q., Sun, T., Hu, Y., Zhang, W., & Ning, Y. (2021). Effectiveness of intervention program on the change of glycaemic control in diabetes with depression patients: A meta-analysis of randomized controlled studies. *Primary Care Diabetes, 15*(3), 428-434. https://doi.org/10.1016/j.pcd.2021.01.006
3. Schmidt, C. B., van Loon, B. J. P., Vergouwen, A. C. M., Snoek, F. J., & Honig, A. (2018). Systematic review and meta‐analysis of psychological interventions in people with diabetes and elevated diabetes‐distress. *Diabetic Medicine, 35*(9), 1157-1172. https://doi.org/10.1111/dme.13709
4. Winkley, K., Upsher, R., Stahl, D., Pollard, D., Brennan, A., Heller, S. R., & Ismail, K. (2020a). Psychological interventions to improve glycemic control in adults with type 2 diabetes: a systematic review and meta-analysis. *Psychosocial Research, 8*(1), e001150. <http://dx.doi.org/10.1136/bmjdrc-2019-001150>
5. Winkley, K., Upsher, R., Stahl, D., Pollard, D., Brennan, A., Heller, S. R., & Ismail, K. (2020b). Systematic review and meta-analysis of randomized controlled trials of psychological interventions to improve glycaemic control in children and adults with type 1 diabetes. *Diabetic Medicine, 37*(5), 735-746. <https://doi.org/10.1111/dme.14264>
6. Xie, J., & Deng, W. (2017). Psychosocial intervention for patients with type 2 diabetes mellitus and comorbid depression: a meta-analysis of randomized controlled trials. *Neuropsychiatric Disease and Treatment, 13*, 2681-2690. https://doi.org/10.2147/NDT.S116465
7. Tasya, Z., Amiruddin, R., Syam, A., Thamrin, Y. (2021). Psychotherapy intervention diabetes distress in diabetes patients: A systematic reivew. *Enfermeria Clinica, 31*, S756-S760. https://doi.org/10.1016/j.enfcli.2021.09.007

**Computerised CBT**

1. Adeel, A., Kannangara, C., Bharaj, H. S., Basu, A., Green, B., Ogilvie, L., & Carson, J. (2023). The importance of digital mental health interventions to support people with diabetes-related psychological outcomes: a narrative review. *Mental Health & Social Inclusion, 27*(2), 186-191. Doi: 10.1108/MHSI-03-2023-0029
2. Adhikary, D., Barman, S., & Ranjan, R. (2023). Internet-Based Cognitive Behavioural Therapy for Individuals With Depression and Chronic Health Conditions: A Systematic Review. *Cureus, 15*(4), e37822. Doi: 10.7759/cureus.37822
3. Franco, P., Gallardo, A. M., & Urtubey, X. (2018). Web-Based Interventions for Depression in Individuals with Diabetes: Review and Discussion. *JMIR Diabetes, 3*(3), e13. Doi: 10.2196/diabetes.9694
4. Tavares Franquez, R., Del Grossi Moura, M., Cristina Ferreira McClung, D., Barberato-Filho, S., Cruz Lopes, L., Silva, M. T., de Sá Del-Fiol, F., & de Cássia Bergamaschi, C. (2023). E-Health technologies for treatment of depression, anxiety and emotional distress in person with diabetes mellitus: A systematic review and meta-analysis. *Diabetes Research and Clinical Practice, 203*, 110854. Doi: 10.1016/j.diabres.2023.110854
5. Yap, J. M., Tantono, N., Wu, V. X., & Klainin-Yobas, P. (2021). Effectiveness of technology-based psychosocial interventions on diabetes distress and health-relevant outcomes among type 2 diabetes mellitus: A systematic review and meta-analysis. *Journal of Telemedicine and Telecare,1*, 1357633X211058329. <https://doi.org/10.1177/1357633X211058329>
6. Varela-Moreno, E., Carreira Soler, M., Guzmán-Parra, J., Jódar-Sánchez, F., Mayoral-Cleries, F., & Anarte-Ortíz, M. T. (2022). Effectiveness of eHealth-Based Psychological Interventions for Depression Treatment in Patients With Type 1 or Type 2 Diabetes Mellitus: A Systematic Review. *Frontiers in Psychology, 12*, 746217. Doi: 10.3389/fpsyg.2021.746217
7. Vázquez-de Sebastián, J., Ciudin, A., & Castellano-Tejedor, C. (2021). Analysis of Effectiveness and Psychological Techniques Implemented in mHealth Solutions for Middle-Aged and Elderly Adults with Type 2 Diabetes: A Narrative Review of the Literature. *Journal of Clinical Medicine, 10*(12), 2701. <https://doi.org/10.3390/jcm10122701>

**Not a systematic review**

1. Carpenter, R., DiChiacchio, T., & Barker, K. (2018). Interventions for self-management of type 2 diabetes: An integrative review. *International Journal of Nursing Sciences, 6*(1), 70-91. Doi: 10.1016/j.ijnss.2018.12.002
2. Eseadi, C., Ugwu, U. C., Seer-Uke, E., Obi, I. R., Obande-Ogbuinya, N., & Anyaegbunam, E. N. (2021). Clinical Utility of Cognitive-Behavioural Coaching for Adult Patients with Type 2 Diabetes. *International Medical Journal, 28*(1), 33-35.
3. Franquez, R. T., de Souza, I. M., & Bergamaschi, C. d. C. (2023). Interventions for depression and anxiety among people with diabetes mellitus: Review of systematic reviews. *PloS One, 18*(2), e0281376. Doi: 10.1371/journal.pone.0281376
4. Jesse, C. D., Creedy, D. K., & Anderson, D. J. (2019). Effectiveness of psychological interventions for women with type 2 diabetes who are overweight or obese: a systematic review protocol. *JBI Database of Systematic Reviews & Implementation Reports, 17*(3), 281-289. Doi: 10.11124/JBISRIR-2017-003589
5. Leite, R. G. O. F., Banzato, L. R., Galendi, J. S. C., Mendes, A. L., Bolfi, F., Veroniki, A. A., Thabane, L., & Nunes-Nogueira, V. (2020). Effectiveness of non-pharmacological strategies in the management of type 2 diabetes in primary care: a protocol for a systematic review and network meta-analysis. *BMJ Open, 10*(1), e034481. Doi: 10.1136/bmjopen-2019-034481
6. Visagie, E., Deacon, E., & Kok, R. (2023). Exploring the role of CBT in the self-management of type 2 diabetes: A rapid review. *Health SA = SA Gesondheid, 28*, 2254. Doi: 10.4102/hsag.v28i0.2254
7. Vlachou, E., Ntikoudi, A., Owens, D. A., Nikolakopoulou, M., Chalimourdas, T., & Cauli, O. (2022). Effectiveness of cognitive behavioral therapy-based interventions on psychological symptoms in adults with type 2 diabetes mellitus: An update review of randomized controlled trials. *Journal of Diabetes & its Complications, 36*(5), N.PAG. doi: 10.1016/j.jdiacomp.2022.108185

**Not CBT**

1. Cheng, L., Sit, J. W. H., Choi, K., Chair, S., Li, X., & He, X. (2017). Effectiveness of Interactive Self-Management Interventions in Individuals With Poorly Controlled Type 2 Diabetes: A Meta-Analysis of Randomized Controlled Trials. *Worldviews on Evidence-Based Nursing, 14*(1), 65-73. Doi: 10.1111/wvn.12191
2. Massey, C. N., Feig, E. H., Duque-Serrano, L., Wexler, D., Moskowitz, J. T., & Huffman, J. C. (2019). Well-being interventions for individuals with diabetes: A systematic review. *Diabetes Research & Clinical Practice, 147*, 118-133. Doi: 10.1016/j.diabres.2018.11.014
3. Mathiesen, A. S., Egerod, I., Jensen, T., Kaldan, G., Langberg, H., & Thomsen, T. (2018). Psychosocial interventions for reducing diabetes distress in vulnerable people with type 2 diabetes mellitus: a systematic review and meta-analysis. *Diabetes, Metabolic Syndrome and Obesity : Targets and Therapy, 12*, 19-33. Doi: 10.2147/DMSO.S179301
4. McBain, H., Mulligan, K., Haddad, M., Flood, C., Jones, J., & Simpson, A. (2016). Self management interventions for type 2 diabetes in adult people with severe mental illness. *Cochrane Database of Systematic Reviews,* (4). Doi: 10.1002/14651858.CD011361.pub2
5. Pascoe, M. C., Thompson, D. R., Castle, D. J., Jenkins, Z. M., & Ski, C. F. (2017). Psychosocial Interventions and Wellbeing in Individuals with Diabetes Mellitus: A Systematic Review and Meta-Analysis. *Frontiers in Psychology, 8*, 2063. Doi: 10.3389/fpsyg.2017.02063
6. Perrin, N., Bodicoat, D. H., Davies, M. J., Robertson, N., Snoek, F. J., & Khunti, K. (2019). Effectiveness of psychoeducational interventions for the treatment of diabetes-specific emotional distress and glycaemic control in people with type 2 diabetes: A systematic review and meta-analysis. *Primary Care Diabetes, 13*(6), 556-567. Doi: 10.1016/j.pcd.2019.04.001

**Includes non-RCTs**

1. Kanapathy, J., & Bogle, V. (2019). The effectiveness of cognitive behavioural therapy for depressed patients with diabetes: A systematic review. *Journal of Health Psychology, 24*(1), 137-149. Doi: 10.1177/1359105317713360
2. Kok, J. L. A., Williams, A., & Zhao, L. (2015). Psychosocial interventions for people with diabetes and co-morbid depression. A systematic review. *International Journal of Nursing Studies, 52*(10), 1625-1639. Doi: 10.1016/j.ijnurstu.2015.05.012

**Not T1DM/T2DM**

1. Li, Y., Storch, E. A., Ferguson, S., Li L., Buys, N., & Sun, J. (2022). The efficacy of cognitive behavioral therapy-based intervention on patients with diabetes: a meta-analysis. *Diabetes Research and Clinical Practice, 189*, 109965. https://doi.org/10.1016/j.diabres.2022.109965

**Third-wave CBT**

1. Yang, X., Li, Z., & Sun, J. (2020). Effects of cognitive behavioral therapy-based intervention on improving glycaemic, psychological, and physiological outcomes in adult patients with diabetes mellitus: A meta-analysis of randomized controlled trials. *Frontiers in Psychiatry, 11*, 711. <https://doi.org/10.3389/fpsyt.2020.00711>

**Below 18 years (>10% of studies)**

1. Efthymiadis, A., Bourlaki, M., & Bastounis, A. (2022). The effectiveness of psychological interventions on mental health and quality of life in people living with type 1 diabetes: a systematic review and meta-analysis. *Diabetology International, 13*(3), 513-521. Doi: 10.1007/s13340-021-00564-9
2. Resurrección, D.,M., Navas-Campaña, D., Gutiérrez-Colosía, M.,R., Ibáñez-Alfonso, J.,A., & Ruiz-Aranda, D. (2021). Psychotherapeutic Interventions to Improve Psychological Adjustment in Type 1 Diabetes: A Systematic Review. *International Journal of Environmental Research and Public Health, 18*(20). Doi: 10.3390/ijerph182010940

**Secondary analysis**

1. Upsher, R., Onabajo, D., Stahl, D., Ismail, K., & Winkley, K. (2021). The Effectiveness of Behavior Change Techniques Underpinning Psychological Interventions to Improve Glycemic Levels for Adults With Type 2 Diabetes: A Meta-Analysis. *Frontiers in Clinical Diabetes and Healthcare, 2*, 699038. Doi: 10.3389/fcdhc.2021.699038
